# Polio‐philanthropy in Africa: A narrative review

**DOI:** 10.1002/hsr2.1339

**Published:** 2023-06-14

**Authors:** Jimoh Amzat, Oliver Razum, Kehinde K. Kanmodi

**Affiliations:** ^1^ Department of Sociology Usmanu Danfodiyo University Sokoto Nigeria; ^2^ Department of Sociology University of Johannesburg Johannesburg South Africa; ^3^ Department of Epidemiology and International Public Health, School of Public Health Bielefeld University Bielefeld Germany; ^4^ Faculty of Dentistry University of Puthisastra Phnom Penh Cambodia; ^5^ Cephas Health Research Initiative Inc. Ibadan Nigeria

**Keywords:** Africa, global health, health promotion, narrative review, philanthropy, polio

## Abstract

**Background and Aim:**

Polio eradication efforts including polio‐philanthropy have been coordinated and sustained since 1988, with the introduction of the Global Polio Eradication Initiative (GPEI). The polio fight is sustained in the name of evidence‐based benevolence or beneficent philanthropy from which Africa has benefited immensely. With the recorded polio cases as of 2023, more efforts and funds are required to eradicate polio. Hence, it is not yet “Uhuru.” Using the Mertonian lens, this study examines polio‐philanthropy in Africa, its unintended consequences, and crucial dilemmas, which could impact the polio fight and polio‐philanthropy.

**Methods:**

This is a narrative review that relies on secondary sources obtained through a thorough literature search. Only studies published in English were utilized. The study synthesized relevant literature in line with the study objective. The following databases were consulted: PubMed, philosopher's index, web of knowledge, Google Scholar, and Sociological Abstracts. Both empirical and theoretical studies were utilized for the study.

**Results:**

Despite significant achievements, the global initiative has shortcomings when examined through the Mertonian lens of manifest and latent functions. The GPEI sets a unilinear goal within multiple challenges. The activities of the philanthropic giants manifest in disempowering rigor, multisectoral neglect, and parallel (health) systems, sometimes, inimical to the national health system. Most philanthropic giants often operate vertically. It is observed that, apart from funding, the last phase of polio‐philanthropy will be defined by some crucial factors, the 4Cs: Communicable disease outbreaks, Conflict, Climate‐related disasters, and Conspiracy theory, which could impact the prevalence or resurgence of polio.

**Conclusion:**

The polio fight will benefit from the persistent drive to reach the finish line as scheduled. The latent consequences or dysfunctions are general lessons for GPEI and other global health initiatives. Therefore, decision‐makers should calculate the net balance of consequences within global health philanthropy for appropriate mitigation.

## 1 INTRODUCTION

Polio has been a major global health issue for more than a century. Unfortunately, it will be a crucial health discourse beyond 2023. Viral diseases (such as polio, Ebola, Zika, and coronavirus disease 2019 [COVID‐19]), which characteristically relapse and re‐emerge, have been a hard nut to crack within the global health space. Hence, polio is still a significant public health challenge. Unvaccinated children have the highest risk of polio, which could attack their nervous system leading to paralysis. The major transmission route, among people, is through nasal and oral secretions, but more commonly through contact with contaminated feces.^1^ The concern that polio poses might remain for some time despite the drive to eradicate it. This is not to undermine global health philanthropic efforts to eradicate the disease. The concern is informed by the pitfalls experienced over the years. There is, however, the hope of a glorious triumph over polio through the global eradication efforts.

Although unmet deadlines raise the fear that polio might be a test for global health philanthropy, polio eradication efforts constitute significant evidence of philanthropic benevolence in global health. There had been significant triumphs in the eradication of smallpox and other diseases. Some were in the Global North; some extended to the Global South and Africa, in particular. The case of malaria is significant in demonstrating global health inequalities in disease eradication targets and policies. Malaria still constitutes a significant health problem in Africa and some other parts of the Global South, but it is a global health problem because of the sustained threat to the Global North. An infection often constitutes a global health problem, once it still exists, as protectionism appears to be the basis of making the eradication of polio and other infectious diseases a global concern.

The concerted efforts to eradicate polio have been relatively mounting and sustained over several decades. It is common to celebrate the certification of “zero case” as polio‐free at national or continental levels, with Asia and Africa almost reaching the landmark. The status of polio in Africa status has fluctuated many times, with the possibility of a resurgence. This signifies that the polio fight will be protracted going beyond the reasonable future, due to repeated “heartbreaks” (threats and resurgence). However, the good news is that the fight is sustainable as global health efforts against polio have been strengthened in the name of evidence‐based benevolence, which is easier represented as beneficent philanthropy. The only difference is that beneficent philanthropy considers the possibility of risk even in a “good” action. “Doing good according to vulnerability” is the hallmark of beneficent philanthropy. Specifically, in the polio fight, the global strategic alliance mobilizes both material and nonmaterial resources to stop polio transmission. This is one of the cases of global health support signifying beneficent philanthropy.

In spite of the foregoing argument concerning beneficent philanthropy, with a significant focus on the Global South, especially Africa, how has the polio fight fared? How much has been committed to the fight globally and what amount will be required to stop polio? What are the crucial dilemmas, which could impact the polio fight and polio‐philanthropy? It is essential to evaluate or assess the state of the polio fight in Africa. It is important to examine whether it has been a resounding success, like the global fight against smallpox. It is imperative to think of the efforts in the fight against polio in light of philanthrocapitalism. This study gets inspiration to examine polio‐philanthropy in Africa within the realm of Mertonian thinking and evaluation.^2^ Irrespective of how critical the discourse might be, Africa has “benefited” immensely from global health efforts against polio. More importantly, however, the relics of the polio fight come with essential lessons for global health. But conceptually, “benefit” can be conceived in light of possible latency or dysfunctions (using the Mertonian lens) inherent in all human efforts. This discourse of inherent latency is typically monikered as a “critical approach”, but at least, its relevance as a lens is indubitable. There is always the “other considerable side.” Some pertinent questions have been raised earlier, which constitute the crux of this article. The article will also assess whether it is Uhuru or not, that is, whether the end of polio has come or not.

## 2 LITERATURE SOURCES

This study is a narrative review that relies on secondary sources obtained through a thorough literature search using several relevant search terms, such as “Polio,” “acute flaccid paralysis,” “AFP,” “funding,” “eradication,” and “Africa.” The literature utilized included published books, journal papers, monographs, and so on. Only studies published in English were consulted. The study synthesized relevant literature in line with the study objective. The following databases were sources of the literature utilized: PubMed, philosopher's index, web of knowledge, Google scholar, and Sociological Abstracts. Both empirical and theoretical papers were utilized for the study.

## 3 THE STATE OF POLIO AND POLIO‐PHILANTHROPY IN AFRICA

The earlier industrialized societies (especially the late 1800s) were marked by the prevalence of polio which paralyzed thousands of children. Polio was brought under significant control after the discovery of a vaccine in the 1950s. The vaccine coverage was later extended to the developing world in the 1970s. Despite the control efforts in the late 1980s, it was reported that polio paralyzed more than a thousand children every day^3^—it was indeed a disastrous global infectious disease of the modern era. While there have been a series of efforts, including the global efforts by Rotary International (around 1985) to combat polio, it was not until 1988 that the World Health Organization (WHO), through its 41st General Assembly, committed to eradicating polio by the year 2000.^4^, ^5^ Hence, the Global Polio Eradication Initiative (GPEI) was established in 1988. Three years later (1991), wild polio was in its last eradication phase in the Americas; by 1994, the Americas were polio‐free. With the continued vaccination program and over half a billion children vaccinated, the WHO European region was certified polio‐free in the year 2002.

In the 1990s, polio was extremely devastating that it accounted for almost 75,000 paralyzes annually in Africa alone.^6^ The late 1990s marked a significant increase in polio vaccination efforts in the African region, with a high record of vaccine saturation in all African regions, especially within the National Immunization Programs. At this point, the main concern of the protectionist approach (vaccinating them to protect us) was that some African countries were responsible for the exportation of wild polio to Western countries. Hence, there was the intensification of the eradication program, with the introduction of the CORE Group Polio Project (CGPP) in 1999. The CGPP is a multipartner and multicountry global health initiative meant to strengthen the host country's surveillance and response system through the provision of technical and financial support. The most significant aspect of the initiative was the strong consideration of grassroots partnership (not previously considered) in the fight against polio. The CGPP initiative significantly improved the grassroots’ acceptability of the immunization program, which before then, was on the verge of collapse. There were many negative events (in the form of misconceptions and community resistance) in Nigeria and a few other countries that prompted a rethink in the late 1990s about local concerns, including health systems and public trust in the polio eradication initiative.

The dawn of the millennium (year 2000s) marked a significant milestone in the journey to “zero” in the global polio incidence, especially in a few countries of the Global South. The WHO Western Pacific and Europe were certified polio‐free in 2000 and 2002, respectively. Another significant initiative was the synchronized Immunization Campaigns/Days, a mass immunization supplementary strategy meant to complement the existing routine immunization program, the largest vaccine program implemented, especially in about 23 African countries (including Benin, Cameroon, Chad, Côte d'Ivoire, Democratic Republic of Congo, Guinea, Liberia, Mali, Niger, Nigeria, and Sierra Leone, among others). The program targeted all children under 5 years of age for door‐to‐door vaccination and involved thousands of volunteers. This demonstrated the resolve of African countries to “Kick Polio Out of Africa”; it was another initiative synchronized with the GPEI in Africa.

The concerted efforts also demonstrated the functions of evidenced‐based beneficence reflected in the GPEI. Despite the missed target of polio eradication by the year 2000, the journey to zero polio continues with resounding keenness and insistence on a world without polio. By 2003, polio was only reported in six countries (Afghanistan, Egypt, India, Niger, Nigeria, and Pakistan), a decline from 125 countries in 1988. By 2006, there were only four countries (Afghanistan, India, Nigeria, and Pakistan) left.^7^ By 2019, there was a remarkable reduction in the number of countries with polio cases to just two (Afghanistan and Pakistan), after Africa was certified polio‐free.^8^, ^9^ Unfortunately, the COVID‐19 pandemic cut short the celebration following some new cases of polio as of January, 2023.

Ekwebelem et al.^10^ described the polio decline as an unprecedented result of concerted efforts—a decline “in the number of confirmed wild polio cases from an estimated 350,000 to only 138 cases at the end of 2020.” The figure included both cases of wild polio and circulating vaccine‐derived poliovirus (cVDPV2). Then, there was the coronavirus of 2019 (COVID‐19) pandemic resulting in the disruption of health systems in several countries.^11^ It was bad news for all global health initiatives, including GPEI. *The Lancet* ^12^ described it as falling at the final hurdle to the finish line. It is unfortunate, but not surprising, that a possible polio resurgence was imminent in Africa,^9^ with cases of wild poliovirus (WPV) reported in Malawi and Mozambique, apart from over 600 cases of cVDPV2.^13^ However, before the COVID‐19‐induced polio vaccination disruption, the problem of resurgence had always been there.

## 4 THE PROBLEM OF REINTRODUCTION AND VACCINATION COVERAGE

The year 2005 marked a significant improvement and a resurgence or re‐emergence of polio in countries previously declared polio‐free. The Centers for Disease Control and Prevention mentioned that 21 previously polio‐free countries, mostly in Africa (including Burkina Faso, Cameroon, Chad, Ethiopia Ghana, Guinea, Mali, and Togo) and in Asia (Saudi Arabia and Yemen), reported imported cases of WPV type 1, specifically from the remaining six polio‐endemic countries (primarily Nigeria) where WPV was endemic.^14^ The threat of imported cases has always been a major global issue in respect of infectious diseases. Countries bordering polio‐endemic areas have a higher risk of sporadic importations.^15^ Hence, every country should improve its polio importation preparedness. The multicountry spread underscores the immunity gaps and weak vaccination response among children in the affected countries. The threat of possible global WPV spread or importation will continue to remain until there is total polio eradication. Globalization and international connectedness will continue to pose risks for the re‐emergence of certain diseases, including WPV. Hence, surveillance systems and immunization programs must be strengthened and sustained to keep track of the possible spread or re‐emergence of WPV.

The most significant measure is to improve vaccination coverage, which is partly marred by vaccine hesitancy and, recently, by the COVID‐19 pandemic. The recommendation suggests a minimum of 95% vaccination coverage of children in endemic countries to prevent the importation and re‐emergence of WPV. There is still a significant polio vaccination gap—in terms of incomplete vaccination and zero‐dose vaccination among under‐five children, especially in sub‐Saharan Africa (SSA). For instance, a study analyzed the National Demographic Health Survey of 25 countries in SSA and reported that full vaccination coverage (not only for polio) ranged from 24% in Guinea to 93% in Rwanda, with some socioeconomic determinants responsible for coverage inequalities.^16^ The analysis used prepandemic data and the pandemic might have, even if slightly, adversely affected the coverage. Low vaccination coverage, such as the rate observed in Guinea, and observed coverage inequalities pose significant polio risks to the unvaccinated, which could also facilitate possible mutation and reintroduction to other countries. In spite of the situation, it is important to acknowledge the remarkable achievement in terms of vaccination coverage and imminent eradication due to polio‐philanthropy.

## 5 THE STATE OF POLIO‐PHILANTHROPY

The polio‐philanthropic journey has been a marathon, not a sprint. Since 1988, there have been financial and technical commitments to ensure people live in a polio‐free world.^17^ Some of the most significant achievements include the eradication of two (Types 2 and 3) of the three strains of the WPV, which saved about 20 million people from paralysis and reduced the burden by 99%, among others.^17^ All credit should go to the philanthropy of multilayered donors and partners to deliver polio vaccines across the world. Although much has been achieved, the last lap might be protracted and challenging due to several factors, including intermittent disruption as a result of conflict and inadequate health infrastructure in many underserved communities. The GPEI's multilayered donors include the national governments, Rotary International, WHO, the US Center for Disease Control and Prevention and the United Nations Children's Fund (UNICEF), the Bill & Melinda Gates Foundation, and Gavi, the Vaccine Alliance. All these donors have been on the frontline mobilizing both human and nonhuman resources to eradicate polio.^17^

Since the launch of the robust polio eradication program in the form of GPEI in 1988, slightly over US$18 billion has been expended through the multilayered and collaborative partnership championed by the WHO and UNICEF.^18^ There have been tremendous intervention activities in over 70 countries. Table 1 shows the top five donors in various categories. This is not to underrate donors with lower donated amounts as every cent counts. The G7 countries (Germany, France, Canada, Italy, Japan, the United Kingdom, and the United States) have been major stakeholders in providing the required support in the fight against polio. Other non‐G7‐The Organization for Economic Cooperation and Development (OECD) countries including Norway, the Netherlands, and Australia have been consistent over the years in providing resources to the GPEI. The United Arab Emirate, Saudi Arabia, and Malaysia top the donors from the Global South, whereas India, Nigeria, and Bangladesh provided the highest domestic resources, mostly in the form of in‐kind contributions—time spent by volunteers, health workers, and others in the implementation of supplementary immunization activities (SIAs). The Bill and Melinda Gates Foundation and the Rotary International led the private sector, in terms of donations to the GPEI. The global concerted efforts through the release of funds have paid off in many respects, but not without some latent consequences, which will be examined later. With the recorded cases in 2023, more efforts and funds are required to reach the finish line.

**Table 1:** Top five donors to GPEI (1985–2020) in various categories (US$ million).

G7 countries		Non‐G7 OECD countries		Other donor countries	
USA	3780.62	Norway	302.33	UAE	109.25
UK	1710.84	Netherlands	113.48	Saudi Arabia	34.51
Germany	730.11	Australia	106.46	Russia	33.00
Canada	633.96	Demark	37.46	Oman	3.20
Japan	576.05	Sweden	30.83	Malaysia	20.58

Private sector/NGO		Multilateral sector		Domestic resources	
Bill & Melinda Gates Foundation	4221.82	World Bank Credits to India	501.71	India	1321.68
Rotary International	2161.08	World Bank Investment Partnership for Polio	411.92	Nigeria	249.96
National Philanthropic Trust/Private Philanthropists	701.33	Islamic Development Bank/Loan/Government of Pakistan	427.02	Bangladesh HNPSP Pooled Funds	99.84
Crown Prince of Abu Dhabi	89.08	UNICEF Regular & Other Resources	262.60	Indonesia	57.90
United Nations Foundation	52.23	Gavi, Vaccine Alliance/IFFIm	182.50	Angola	54.03

*Note*: The Global Polio Eradication Initiative (GPEI), 2022.^18^

Abbreviations: EU, European Union; IFFIm, International Finance Facility for Immunization; NGO, nongovernment organization; OECD, The Organization for Economic Cooperation and Development.

## 6 POLIO‐PHILANTHROPY: MOVING FORWARD TO THE GOAL (2022–2026)

Despite the confounding challenges and the repeated missed deadlines, there is a new 5‐year plan and costs to meet the new endline, which should be, purportedly, the last phase in the polio eradication efforts. The new strategy is estimated to cost a minimum of US$ 4.5 billion.^17^ The most critical observation is that what is required is about 30% of what has been contributed in the last 40 years. Raising the funds might be challenging, but it is realizable with the commitment from global development partners. With the Polio Eradication Strategy 2022–2026, the GPEI is geared toward delivering on the promise of eradication, notwithstanding the current programmatic and epidemiological challenges and the 4Cs (Communicable disease outbreaks, Conflict, Climate‐related disasters, and the Conspiracy theory; see Figure 2). The goals remain to stop poliovirus transmission in endemic countries and stop cVDPV transmission and prevent outbreaks in nonendemic countries. The timeline, which functions as a budget and planning tool, is subject to review from time to time (Table 2).

**Table 2:** Estimated cost of various eradication timelines.

Eradication timeline	Projected 5‐year cost (US$)
Interrupt WPV transmission in 1 year and certify in 4 years	4.5 billion
Interrupt WPV transmission in 2 years and certify in 4 years	5.1 billion
Interrupt WPV transmission in 3 years and certify in 4 years	5.5 billion
Interrupt WPV transmission in 5 years and certify in 8 years	6.2 billion

*Note*: WHO, 2021, p. 38.^19^

Abbreviations: WHO, World Health Organization; WPV, wild poliovirus.

The important milestone will be the year the world records its last case, and then, a possible global polio‐free certification after 3 years. Table 2 shows four possible modeling scenarios with varying eradication timelines. Although the WHO assumes that the second scenario of the last case in 2023 and certification in 2026 is possible, available evidence and new cases show it might not be possible. With the antecedents and trends of the polio fight, it is better to adopt another scenario, the last case in 2025 with certification in 2028, and work assiduously towards it. The only problem is whether the funds, an estimated $5.5 billion will available or not. Hence, it is not yet “uhuru.” Significantly, the GPEI has been a laudable drive, but some latent functions or dysfunctions of the polio‐philanthropy are observable.

## 7 LATENCIES IN POLIO‐PHILANTHROPY IN AFRICA

In praise of philanthropy, it is a transformative gesture to tackle the unmet needs of the underserved. Most times, beneficent philanthropy comes with some costs, which are often ill‐defined within the philanthropic framework. There is always the overwhelming focus on target outcomes, which is herein described as the manifest function of philanthropy. The global health space has these two players: the givers and the takers. At the manifest level, for those that carry the pains or burden of others through philanthropy, they do, owing to their obligation in global health that no country should be left behind, in this case, in the polio fight. The global connectivity shows a common destiny, especially as a result of a probable spark, spread and resurgence of infectious diseases. At the latent level, there are inherent shortcomings or dysfunctions, sometimes, unintended or uncalculated. Merton^2^ observed that all social processes are not inherently and absolutely good; the outcomes “depend” on circumstances and trajectories. Hence, there are less apparent, sometimes unintended, and often neglected functions of social institutions and processes. Social investments, interventions, and philanthropy could be dysfunctional—perhaps not entirely but meaningfully to warrant a rethink in terms of the implementation process: contents, prioritization and possible outcomes.

It is the “norm” to recognize positive and measurable outcomes, while the silent dysfunctions wreak in bits. The first primary dilemma is that the health space contracts, thereby evolving into a dependency. Hence, the latent dysfunction of philanthropy is the possible weakened/lessened adaptation and adjustment mechanisms in the health system. External influence/interventions sometimes erode or lessen the (political) sense of responsibility, which, in the long run, could reduce the health system's vulnerability in terms of disease prevalence and reduced strategy.

Philanthropy is a function of governmentality; hence, it is value‐ and interest‐laden—contaminated by the donors’ ideologies. It is a hegemonic tool in global health for self‐protection and crisis mitigation. What is paramount is that the interest of the givers supersedes all other “altruistic” considerations. For instance, there is gradual involvement of purely for profit‐private organizations and consulting firms in health interventions. Hence, there is nothing like total altruism but imbued hegemonic stance and inputs into reform packages favoring certain contents with self‐effacement which question altruism and accountability.^20^, ^21^ The global health space is characterized by multi‐actor with somehow hidden or open power inequalities. The value‐laden donations of super‐philanthropists have the tendency of disempowering or marginalizing the recipients,^20^ due to the privilege (the super‐donors have) to set the agenda. Youde^20^ described the situation as an overriding but not coercive power to frame and control health discourse. In some, it is a case of passive coercion, when the recipients have little or no power to significantly influence the agenda.

The argument is not about the diminution of political power for states, but about indirect consequences, of adhering or complying with alien conditionalities and agendas to accessing and utilizing the aid. It could, at the manifest but more at the latent level, be about changing the logic of health governance in terms of priority setting and resource allocation. The aforementioned produces a passive recipient subjected to a wider redefined and reconfigured responsibility within the “collaborative” alliance.^20^ Youde^20^ noted that although philanthropists are not neutral actors or apolitical actors, their influences and biases are often unduly unobserved. Therefore, decision‐makers should constantly calculate the net balance of consequences within global health philanthropy for appropriate mitigation.

Polio‐philanthropy cannot be regarded as a non‐performing aid in terms of the goal; there has been a drastic reduction in the prevalence of polio. However, there are, most times, latent consequences as a result of the control strategies which undermine the health systems of the Global South. Although the program has been a private–public partnership, with the inclusion of non‐western nations, the GPEI is somewhat portrayed as philanthrocapitalism. In its real sense, “there is no free lunch” in a capitalist economy. However, where there seems to be a free lunch, it is only that the cost is not obvious. Embedded in philanthrocapitalism, are latent costs, which might undermine the rights, finance and development of receiving nations.^22^ The program comes with intervention fierceness ensuring deeper penetration and saturation which account for the achieved outcome—that is, over 99% reduction in polio incidence. The ultimate costs include an altered sense of responsibility, balance of power, and induced vulnerability within the health systems.

**Figure 1:**
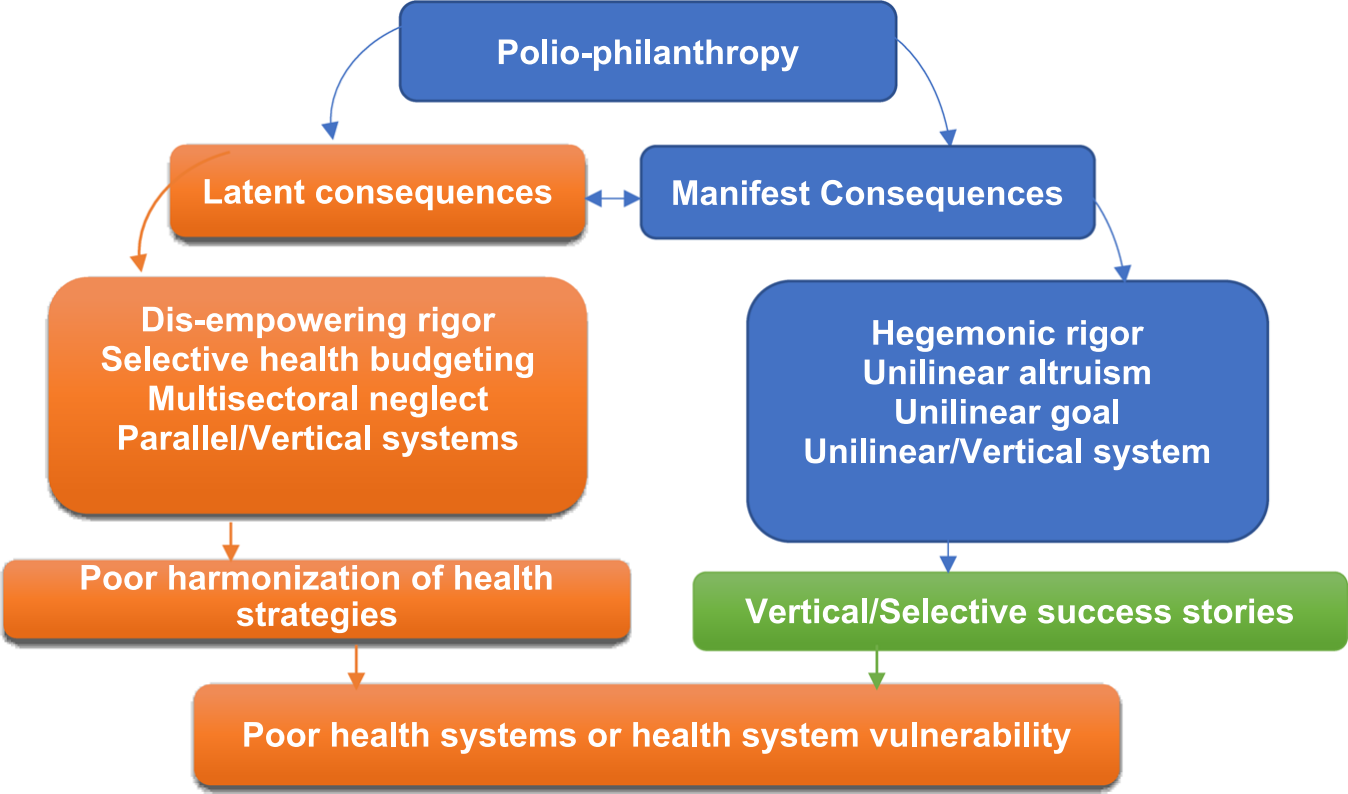
Latent consequences of polio‐philanthropy.

Figure 1 presents a concise framework for the latent consequences of polio‐philanthropy, which can be applied to other global health philanthropies, as they all mirror a similar idiosyncrasy. The figure shows that polio‐philanthropy has manifest and latent consequences. Although “unintended” or “unplanned,” they are meaningful for the health systems. However, that it is unplanned does not exonerate the philanthropists from responsibility. Health philanthropy is fraught with hegemonic rigors as well as unilinear altruism and goal which lead to a unilinear system. Within one health system are many unilinear systems, which generate selective/vertical success stories as witnessed in the eradication of smallpox, and the near eradication of polio.

Moreover, the GPEI sets a unilinear goal within multiple challenges. The activities of the philanthropic giants manifest in disempowering rigor, selective health budgeting, multisectoral neglect, and parallel (health) systems. Such parallel systems/bodies are, sometimes, inimical to the national health system.^23^ Most philanthropic organizations or structures operate vertically without properly aligning with the national health system. Amzat and Razum^24^ observed that the vertical approach presents a disease‐specific and top‐down agenda according to the funder's ideology. A vertical or standalone approach is narrow and targets specific diseases, thereby neglecting others even where they are significant health problems. The vertical approach results in a duplication of efforts, disproportionate funding, and ineffectual surveillance systems^25^ instead of the horizontal approach which comes with the Integrated Disease Surveillance and Response (IDSR).

Although the core of the health system's goal is the horizontal or multisectoral approach to maximize efficiency, there is always the presence of strong vertical programs on diseases like polio, malaria, HIV and tuberculosis—the result is a nonintegrative or nonmultisectoral system.^25^, ^26^ The consequent danger (observable in the GPEI) is the skewed concern with vaccine provision, inter‐governmental relations and considerably low levels of health sector‐wide support and civil partnerships.^27^ In the skewness, the social determinants of health and water, sanitation, and hygiene (WaSH), which are strongly connected to polio emergence have no space or place.

The vertical approach creates separate resources for specific purposes, resulting in poor harmonization of health priorities, strategies, surveillance, and response systems (Figure 1). The vertical approach is a product of the global health initiative in the form of purposive development assistance for health.^24^ Beyond these latent consequences, there are other confounding dilemmas that could adversely impact the polio eradication timeline in Africa.

## 8 CONFOUNDING DILEMMAS IN THE POLIO FIGHT AND PHILANTHROPY

**Figure 2:**
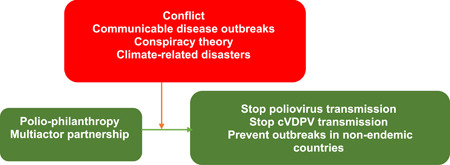
Four significant factors militating against the polio fight.

The future of polio‐philanthropy will be defined by several confounding dilemmas, specifically, the 4Cs (Figure 2) all of which could have a significant effect on the prevalence or resurgence of polio and polio‐philanthropy. There have been some disruptions in the polio vaccination programs. The most recent was as a result of the COVID‐19 pandemic. The pandemic was a significant drawback for all vaccination programs, especially as a result of the lockdown and health services disruption in many countries. Burkholder^28^ observed that the pandemic disrupted efforts to deliver essential health services worldwide. The outcomes include the programmatic and epidemiologic effects on the polio fight, especially the immediate suspension of the SIAs and the house‐to‐house vaccination program. The pandemic is one of the factors responsible for the resurgence of polio in some previously certified polio‐free African countries. This practically extended the end‐line for the polio eradication program by, at least, 4 years. It is worth noting that there was a drastic push to deliver polio vaccine and continue polio surveillance. The resilience and program modifications serve to limit transmission and resurgence to a large extent through some significant operational adaptations and flexible planning. Therefore, communicable disease outbreaks (including Ebola and cholera, among others) could affect the next phase in the polio fight.

Ali and Hamid^29^ noted that other local challenges such as natural disasters have over the years constituted a challenge to the vaccination programs by occasionally disrupting health services or deprioritizing vaccination programs. They cited examples of climate‐related disasters such as “super floods.” In most African countries, adverse weather events have been a major challenge to public health, particularly vaccination programs. Mahmud et al.^30^ observed that climate‐related disasters adversely affect the dynamics of many communicable diseases, including vaccine‐preventable ones. Such disasters disproportionately precipitate disease by altering the burden and geographic coverage of vaccination. Any adverse events will affect the global polio eradication goal, among other goals. Disaster risks, in the form of extreme weather events, happen occasionally but wreak havoc affecting millions of people.^31^, ^32^

Drought, a protracted dry period in the climate cycle, is another climate‐related event that has adverse effects on child vaccination programs.^33^ In the face of severely limited access to water and food, a vaccine might be regarded as a secondary need. Disasters could amount to a pathetic trade‐off between vaccines and other basic needs (especially food and WaSH). Where vaccines are available, their positive effects might be subsided within the population when faced with disasters such as drought and flooding. Nagata et al.^33^ observed that drought was adversely associated with childhood immunization completion, including polio vaccinations in SSA. It hinders vaccination coverage and completion as a result of food insecurity and frequent migration (in search of food), which are generally associated with poor health outcomes. Drought affects many countries in Africa (including Sudan, Somalia, South Sudan, Tanzania, and Uganda, among others). The result is reduced agricultural production, which is the main economic activity in the region. As drought leads to financial and food insecurity, it induces child malnutrition, limits access to healthcare and leads to poor health outcomes.^34^, ^35^

Furthermore, conflict and wars pose tremendous challenges to vaccination programs. Unfortunately, many African countries (such as Burkina Faso, Cameroon, Chad, DR Congo, Libya, Mali, Mozambique, Niger, Nigeria, Somalia, and South Sudan) are ravaged by conflict/wars. Conflict leads to geographical inaccessibility to children who require vaccines. Conflict also disrupts vaccination programs due to insecurity and mass displacement problematizing the coverage of vaccination programs which can subsequently limit population immunity, thereby increasing vulnerability to outbreaks of polio and other vaccine‐preventable diseases.^27^, ^36^, ^37^, ^38^ Sato^37^ focused on the terrorism‐ravaged northeast Nigeria and observed a strong negative effect of conflict events on the likelihood of vaccination. The ultimate conclusion was that armed conflicts/insurgency had a devastating effect on the vaccination of young children born during conflicts in the region. Grundy & Briggs^27^ reported similar findings, which show that conflict was responsible for the destruction of health infrastructure and depleted human resources responsible for infrequent outreach services, especially SIAs, in 16 countries (including Ethiopia, Kenya, Uganda, Nigeria, Pakistan, Somalia, Sierra Leone, Central African Republic, Sudan, and South Sudan among others). Insecurity results in pockets of low coverage and disease outbreaks at different times in the conflict‐affected areas. Therefore, the conflict situation in the last phase of the polio fight will determine if and when the eradication goal will be reached.

The polio vaccination program has been marred by rumors and mis‐/dis‐information, or in short, infodemic—all embedded in the conspiracy theory, mostly due to insufficient civil/public engagement. This accounted for the initial vaccine hesitancy or resistance, which has drastically reduced but not completely subdued. The initial conspiracy theory was that polio vaccine was contaminated with antifertility substances meant to reduce the population of some low‐income countries, especially in Muslim‐dominated regions.^39^ Later, polio vaccine hesitancy was about inadequate trust in vaccine quality, adverse side effects, and the influence of negative social media messages.^40^, ^41^, ^42^ Infodemic, the abundance of accurate and inaccurate information, especially on the social media, fuels rumors and undermines public trust in vaccination efforts. Hence, infoveillance, public engagement, and health communication are key to correcting misinformation and reducing vaccine skepticism.

## 9 CONCLUSION

Polio eradication efforts have been coordinated and sustained since 1988, with the introduction of the GPEI. Africa has benefited immensely from the global health efforts against polio. Slightly over US$18 billion has been expended through the multilayered and collaborative partnership championed by the WHO and UNICEF. With the recorded cases as of 2023, more efforts and funds (an estimated $4.5–$5.5 billion) are required to reach the finish line. Hence, it is not yet Uhuru. Apart from funding, reaching the polio eradication goal will be affected by some factors, including the 4Cs, which could significantly affect the prevalence or resurgence of polio.

Despite some significant achievements, the global initiative has some shortcomings when examined through the Mertonian lens of manifest and latent functions. The GPEI sets a unilinear goal within multiple challenges. The activities of the philanthropic giants manifest in disempowering rigor, multisectoral neglect, and parallel (health) systems, which are sometimes inimical to national health systems. The observed shortcomings and confounding factors are relics from the polio fight, which should be considered in other global health efforts. There is strong optimism that with sustained resources (both human and nonhuman), the end of polio only remains a few years.

## AUTHOR CONTRIBUTIONS

**Jimoh Amzat**: Conceptualization; data curation; formal analysis; funding acquisition; investigation; methodology; project administration; resources; software; supervision; validation; visualization; writing—original draft; writing—review & editing. **Oliver Razum**: Funding acquisition; resources; validation; writing—review & editing. **Kehinde K. Kanmodi**: Funding acquisition; writing—review & editing.

## CONFLICT OF INTEREST STATEMENT

Kehinde K. Kanmodi is an Editorial Board member of Health Science Reports and a coauthor of this article. To minimize bias, they were excluded from all editorial decision‐making related to the acceptance of this article for publication. Other authors have no conflict of interest to declare.

## ETHICS STATEMENT

Not applicable. This study did not collect data from human or animal subjects but from an open research repository.

## TRANSPARENCY STATEMENT

The lead author Kehinde K. Kanmodi affirms that this manuscript is an honest, accurate, and transparent account of the study being reported; that no important aspects of the study have been omitted; and that any discrepancies from the study as planned (and, if relevant, registered) have been explained.

## Data Availability

Data sharing is not applicable to this article as no new data were created or analyzed in this study.

## References

[1] WHO [World Health Organization] . Poliomyelitis [Internet]. 2023. Accessed February 21, 2023. https://www.who.int/teams/health-product-policy-and-standards/standards-and-specifications/vaccine-standardization/poliomyelitis

[2] Merton RK . Manifest and latent functions. In: Longhofer W. , ed. Social Theory Re‐Wired. Revised. Second Edition. Routledge; 2016 10.4324/9781315775357

[3] Global Polio Eradication Initiative (GPEI) . History of Polio. 2022. Accessed January 8, 2023. https://polioeradication.org/polio-today/history-of-polio/

[4] Cochi SL , Hegg L , Kaur A , Pandak C , Jafari H . The Global Polio Eradication Initiative: progress, lessons learned, and polio legacy transition planning. Health Aff. 2016;35(2):277‐283. 10.1377/hlthaff.2015.1104 26858381

[5] Razum O , Sridhar D , Jahn A , Zaidi S , Ooms G , Müller O . Polio: from eradication to systematic, sustained control. BMJ Glob Health. 2019;4(4):e001633. 10.1136/bmjgh-2019-001633 PMC6730569 31544903

[6] WHO‐Africa . Africa eradicates wild poliovirus [Internet]. WHO | Regional Office for Africa. 2020. Accessed February 21, 2023. https://www.afro.who.int/news/africa-eradicates-wild-poliovirus

[7] CDC [Centers for Disease Control and Prevention] . Progress toward interruption of wild poliovirus transmission ‐ worldwide, January 2007‐April 2008. MMWR Morb Mortal Wkly Rep. 2008;57(18):489‐494. 18463607

[8] Effiong F , Akanno IP , Anosike UG , et al. Nigeria's polio elimination playbook: lessons to strengthening health systems for other eradicable diseases. Global Biosecurity. 2021;3(1). 10.31646/gbio.127

[9] Uwishema O , Elebesunu EE , Bouaddi O , et al. Poliomyelitis amidst the COVID‐19 pandemic in Africa: efforts, challenges and recommendations. Clin Epidemiol Global Health. 2022;16:101073. 10.1016/j.cegh.2022.101073 PMC9148425 35664665

[10] Ekwebelem OC , Nnorom‐Dike OV , Aborode AT , Ekwebelem NC , Aleke JC , Ofielu ES . Eradication of wild poliovirus in Nigeria: lessons learnt. Public Health Pract. 2021;2:100144. 10.1016/j.puhip.2021.100144 PMC9461633 36101607

[11] Assefa N , Sié A , Wang D , et al. Reported barriers to healthcare access and service disruptions caused by COVID‐19 in Burkina Faso, Ethiopia, and Nigeria: A telephone survey. Am J Trop Med Hyg. 2021;105(2):323‐330. 10.4269/ajtmh.20-1619 34161296 PMC8437171

[12] Lancet T. Polio eradication: falling at the final hurdle? Lancet. 2022;400(10358):1079. 10.1016/S0140-6736(22)01875-X 36183713

[13] Global Polio Eradication Initiative (GPEI) . Polio as of this week, 05 October. 2022. Accessed October 13, 2022. https://polioeradication.org/polio-today/polio-now/this-week/

[14] CDC [Centers for Disease Control and Prevention] . Resurgence of wild poliovirus type 1 transmission and consequences of importation: 21 countries, 2002‐2005. MMWR Morb Mortal Wkly Rep. 2006;55(06):145‐150. 16484977

[15] CDC [Centers for Disease Control and Prevention] . Progress toward interruption of wild poliovirus transmission ‐ worldwide, January 2006‐May 2007. MMWR Morb Mortal Wkly Rep. 2007;56(27):682‐685. 17625496

[16] Bobo FT , Asante A , Woldie M , Dawson A , Hayen A . Child vaccination in sub‐Saharan Africa: increasing coverage addresses inequalities. Vaccine. 2022;40(1):141‐150. 10.1016/j.vaccine.2021.11.005 34794824

[17] WHO [World Health Organization] . Global Polio Eradication Initiative Investment Case ‐2026: investing in the promise of a polio‐free world, 2022.

[18] Global Polio Eradication Initiative (GPEI) . Contributions and Pledges to the Global Polio Eradication Initiative, 1985‐2020. 2022. Accessed January 10, 2023. https://polioeradication.org/wp-content/uploads/2021/07/GPEI_FIN_Historical-Contributions_Journals-Charts_asat_2020-12-31.pdf

[19] WHO [World Health Organization] . Polio Eradication Strategy 2022–2026: Delivering on a Promise, 2021.

[20] Youde J . The role of philanthropy in international relations. Rev Int Studies. 2019;45(1):39‐56. 10.1017/S0260210518000220

[21] Eckl J , Hanrieder T . The political economy of consulting firms in reform processes: the case of the World Health Organization. Rev Int Pol Econ. 2023:1‐24. 10.1080/09692290.2022.2161112

[22] Mcgoey L . No such thing as a free gift: the Gates Foundation and the price of philanthropy. Verso; 2016.

[23] Biesma RG , Brugha R , Harmer A , Walsh A , Spicer N , Walt G . The effects of global health initiatives on country health systems: a review of the evidence from HIV/AIDS control. Health Policy Plan. 2009;24(4):239‐252. 10.1093/heapol/czp025 19491291 PMC2699244

[24] Amzat J , Razum O . Global Health Initiatives in the Global South. Routledge; 2022:186‐208. 10.4324/9781003247975-11

[25] Onwe FI , Okedo‐Alex IN , Akamike IC , Igwe‐Okomiso DO . Vertical disease programs and their effect on Integrated Disease Surveillance and Response: perspectives of epidemiologists and surveillance officers in Nigeria. Trop Dis Travel Med Vaccines. 2021;7(1):28. 10.1186/s40794-021-00152-4 34593034 PMC8483794

[26] Mremi IR , George J , Rumisha SF , Sindato C , Kimera SI , Mboera LEG . Twenty years of Integrated Disease Surveillance and Response in Sub‐Saharan Africa: challenges and opportunities for effective management of infectious disease epidemics. One Health Outlook. 2021;3(1):22. 10.1186/s42522-021-00052-9 34749835 PMC8575546

[27] Grundy J , Biggs BA . The impact of conflict on immunisation coverage in 16 countries. Int J Health Policy Manag. 2019;8(4):211‐221. 10.15171/ijhpm.2018.127 31050966 PMC6499911

[28] Burkholder B , Wadood Z , Kassem AM , Ehrhardt D , Zomahoun D . The immediate impact of the COVID‐19 pandemic on polio immunization and surveillance activities. Vaccine. 2023;41 Suppl 1(21):2. 10.1016/j.vaccine.2021.10.028 PMC8531002 34756614

[29] Ali I , Hamid S . Implications of COVID‐19 and “super floods” for routine vaccination in Pakistan: the reemergence of vaccine preventable‐diseases such as polio and measles. Hum Vaccines Immunother. 2022;18(7):2154099. 10.1080/21645515.2022.2154099 PMC9891673 36573023

[30] Mahmud AS , Martinez PP , He J , Baker RE . The impact of climate change on vaccine‐preventable diseases: insights from current research and new directions. Curr Environ Health Rep. 2020;7(4):384‐391. 10.1007/s40572-020-00293-2 33099754 PMC7585557

[31] Fraser A , Leck H , Parnell S , Pelling M . Africa's urban risk and resilience. Int J Disaster Risk Reduct. 2017;26:1‐6. 10.1016/j.ijdrr.2017.09.050

[32] Shimada G . The impact of climate‐change‐related disasters on Africa's economic growth, agriculture, and conflicts: can humanitarian aid and food assistance offset the damage? Int J Environ Res Public Health. 2022;19(1):467. 10.3390/ijerph19010467 35010724 PMC8744906

[33] Nagata JM , Epstein A , Ganson KT , Benmarhnia T , Weiser SD . Drought and child vaccination coverage in 22 countries in Sub‐Saharan Africa: a retrospective analysis of national survey data from 2011 to 2019. PLoS Med. 2021;18(9):e1003678. 10.1371/journal.pmed.1003678 34582463 PMC8478213

[34] Asmall T , Abrams A , Röösli M , Cissé G , Carden K , Dalvie MA . The adverse health effects associated with drought in Africa. Sci Total Environ. 2021;793:148500. 10.1016/j.scitotenv.2021.148500 34174598

[35] Le K , Nguyen M . Droughts and child health in Bangladesh. PLoS One. 2022;17(3):e0265617. 10.1371/journal.pone.0265617 35312716 PMC8936449

[36] Kennedy J , McKee M , King L . Islamist insurgency and the war against polio: a cross‐national analysis of the political determinants of polio. Glob Health. 2015;11(1):40. 10.1186/s12992-015-0123-y PMC4589183 26420386

[37] Sato R . Effect of armed conflict on vaccination: evidence from the Boko haram insurgency in northeastern Nigeria. Confl Health. 2019;13(1):49. 10.1186/s13031-019-0235-8 31673285 PMC6819527

[38] Maleghemi S , Tegegne AA , Ferede M , et al. Polio eradication in a chronic conflict setting lessons from the Republic of South Sudan, 2010‐2020. Pan Afr Med J. 2022;42(suppl 1):3. 10.11604/pamj.supp.2022.42.1.32922 PMC9474935 36158939

[39] Renne E . Perspectives on polio and immunization in Northern Nigeria. Soc Sci Med. 2006;63(7):1857‐1869. 10.1016/j.socscimed.2006.04.025 16765498

[40] Abbasi FH , Shaikh AA , Mehraj J , et al. Vaccine hesitancy and perceptions of the community about polio in high‐risk areas of Karachi, Sindh, Pakistan. Vaccines. 2022;11(1):70. 10.3390/vaccines11010070 36679915 PMC9866813

[41] Lohiniva AL , Nurzhynska A , Alhassan H , Shetye M , Ayiku P . Understanding factors influencing polio vaccine uptake in Ghana‐developing meaningful community mobilization and engagement strategies in collaboration with religious leaders. Am J Trop Med Hyg. 2022;107(6):1345‐1350. 10.4269/ajtmh.22-0271 36315999 PMC9768250

[42] Yakum MN , Funwie AD , Ajong AB , Tsafack M , Ze LEE , Shah Z . The burden of vaccine hesitancy for routine immunization in Yaounde‐Cameroon: a cross‐sectional study. PLOS Glob Public Health. 2022;2(9):e0001012. 10.1371/journal.pgph.0001012 36962666 PMC10022391

